# Efficacy and safety of the endothelin-1 receptor antagonist macitentan in epicardial and microvascular vasospasm; a proof-of-concept study

**DOI:** 10.1016/j.ijcha.2023.101238

**Published:** 2023-07-16

**Authors:** Rutger G.T. Feenstra, Tijn P.J. Jansen, S. Matthijs Boekholdt, Janet E. Brouwer, Margriet I. Klees, Yolande Appelman, Marianne E. Wittekoek, Tim P. van de Hoef, Robbert J. de Winter, Jan J. Piek, Peter Damman, Marcel A.M. Beijk

**Affiliations:** a Amsterdam UMC, Heart Center, Department of Cardiology, Amsterdam Cardiovascular Sciences, Amsterdam, the Netherlands; b Department of Cardiology, Radboud University Medical Center, Nijmegen, the Netherlands; c Cardiology, HeartLife Clinics, Utrecht, the Netherlands; d Department of Cardiology, University Medical Center Utrecht, Utrecht, the Netherlands

**Keywords:** Vasospastic angina, Microvascular angina, Coronary artery spasm, Macitentan, Proof of concept

## Abstract

**Background:**

Treatment of patients diagnosed with angina due to epicardial or microvascular coronary artery spasm (CAS) is challenging because patients often remain symptomatic despite conventional pharmacological therapy. In this prospective, randomized, double-blind, placebo-controlled, sequential cross-over proof-of-concept study, we compared the efficacy and safety of macitentan, a potent inhibitor of the endothelin-1 receptor, to placebo in symptomatic patients with CAS despite background pharmacological treatment.

**Methods:**

Patients with CAS diagnosed by invasive spasm provocation testing with >3 anginal attacks per week despite pharmacological treatment were considered for participation. Participants received either 10 mg of macitentan or placebo daily for 28 days as add-on treatment. After a wash-out period patients were crossed over to the alternate treatment arm. The primary endpoint was the difference in anginal burden calculated as [1] the duration (in minutes) * severity (on a Visual Analogue Scale (VAS) pain scale 1–10); and [2] the frequency of angina attacks * severity during medication use compared to the run-in phase.

**Results:**

28 patients of whom 22 females (79%) and a mean age of 55.3 ± 7.6 completed the entire study protocol (epicardial CAS n = 19 (68), microvascular CAS n = 9 (32)). Change in both indices of anginal burden were not different during treatment with add-on macitentan as compared to add-on placebo (duration*severity: −9 [−134 78] vs −45 [−353 11], p = 0.136 and frequency*severity: −1.7 [−5.8 1.2] vs −1.8 [−6.2 0.3], p = 0.767). The occurrence and nature of self-reported adverse events were closely similar between the treatment phase with macitentan and placebo.

**Conclusion:**

In patients with angina due to epicardial or microvascular CAS despite background pharmacological treatment, 28 days of add-on treatment with the ET-1 receptor antagonist, macitentan 10 mg daily, did not reduce anginal burden compared to add-on treatment with placebo.

Trial Registrationhttps://trialsearch.who.int/, Identifier: EUCTR2018-002623-42-NL. Registration date: 20 February 2019.

## Highlights

• Treatment of patients diagnosed with angina due to epicardial or microvascular coronary artery spasm is challenging because patients often remain symptomatic despite conventional pharmacological therapy.
• Macitentan is a potent inhibitor of the endothelin-1 receptor and therefore a potential pharmacological therapeutic agent for patients with coronary artery spasm.
• In this prospective, randomized, double-blind, placebo-controlled, sequential cross-over proof-of- concept study, add-on treatment with Macitentan 10 mg daily for 28 days, did not reduce angina burden compared to add-on treatment with placebo.

## 1 Introduction

Approximately 40% of patients undergoing coronary angiography for the evaluation of angina have no obstructive coronary artery disease (ANOCA) [1]. In the many of these patients, the underlying pathophysiology is coronary artery spasm (CAS) [2] . It consists of an exaggerated vasoconstrictive response that can occur on the epicardial or microvascular level of the coronary circulation, yet the pathophysiology is not fully elucidated [3]. Pharmacological treatment of CAS is challenging and patients often remain symptomatic despite pharmacological treatment. Hence, there is a need for pharmacological studies in order to reduce anginal burden and improve quality of life in patients with CAS [4], [5].

Endothelin-1 (ET-1) is a small peptide predominantly released by vascular endothelial cells. Through its paracrine action ET-1 plays an important physiological and pathophysiological role, especially in the regulation of vascular tone [6]. ET-1 induces an extremely potent and long-lasting vasoconstriction. Moreover, ET-1 affects the production of other local mediators of the vascular tone, including NO, prostacyclin, and platelet-activating factor [7]. Other effects of ET-1 include pro-inflammatory actions, mitogenic and proliferative effects, stimulation of free radical formation and platelet activation [8]. Previously, it was shown that peripheral arterioles from patients with proven ischemia and non-obstructive coronary artery disease show enhanced vasoconstriction to ET-1 compared with reference subjects [9]. Finally, ET-1–induced vasoconstriction is insensitive to the current recommended therapy with calcium channel-antagonism [10]. Considering the above, endothelial receptor antagonism (ERA) may be a potential pharmacological treatment strategy in patients with CAS. Thus far, clinical evidence derived from randomized controlled trials from pharmacological therapy with ERA in ANOCA is limited to patients with coronary microvascular endothelial dysfunction [11], while that in patients with CAS is limited to pre-clinical studies and a number of case reports that have documented a beneficial effect [9], [10], [12], [13]. In the randomized, double-blind, cross-over, placebo-controlled proof of concept VERA study, we evaluated the efficacy and safety of the ET-1 receptor antagonist macitentan for the treatment in symptomatic patients due to epicardial or microvascular CAS despite background pharmacological treatment.

## 2 Methods

### 2.1 Study design

The VERA study is a randomized, double-blind, placebo-controlled, sequential cross-over, multicenter clinical proof-of-concept study with the aim to evaluate the efficacy and safety of macitentan versus placebo in symptomatic patients with acetylcholine vasospasm provocation proven CAS despite background anti-anginal therapy. Patients were recruited from three Dutch centers with expertise in treating functional coronary artery disease (Amsterdam University Medical Center, Amsterdam, the Netherlands; HeartLife Clinics, Utrecht, the Netherlands; University Medical Center, Nijmegen, the Netherlands).The protocol was approved by all the local Institutional Review Boards and all patients gave written informed consent. All data were entered into an electronic database (Castor EDC). VERA trial is registered at trialregister.nl (NL7546).

Study patients

Between November 2019 and July 2021, patients at the outpatient clinic with known epicardial or microvascular CAS as assessed by spasm provocation testing were screened. The specific acetylcholine vasospasm protocols used to diagnose CAS have been described previously in detail [14], [15], [16]. Patients were eligible for inclusion if all of the following were present: 1) age ≥ 18; 2) absence of significant obstructive coronary artery disease (defined as stenosis > 50% in an epicardial coronary artery) documented by invasive CAG; 3) anginal symptoms with a frequency of at least 3 times per week despite anti-anginal pharmacological therapy (at least treatment with CCB and/or long-acting nitrates, angiotensin converting enzyme inhibitors or angiotensin receptor antagonists); 4) anginal symptoms for at least 3 months despite optimal anti-anginal therapy, which was at the discretion of the treating cardiologist; 5) able to comply with the study procedures; 6) able to provide written informed consent. The exclusion criteria were: 1) pregnant or nursing women and those who plan pregnancy in the period up to 1 months after the study; 2) women of childbearing potential not using contraception; 3) Contra-indication for macitentan, i.e. patients with active liver disease or severe liver disease (ASAT and/or ALAT > 3x upper limit of normal (ULN)), renal impairment (GFR < 60 ml/min), anemia, use of potent CYP3A4 inducers/inhibitors; and 4) Patients with a limited life expectancy<1 year.

Patients were enrolled at the screening visit after written informed consent was obtained and the inclusion and exclusion criteria were met. Moreover, pharmacological therapy was documented, and clinical examination and laboratory measurements performed. The frequency of angina was evaluated by means of an angina diary during a 28-days run-in phase of the study. After this run-in-phase eligible patients were randomized. Randomization was performed using a computerized randomization tool in Castor. Eligible and consenting patients were block randomized (block size of four and six) to the two groups reflecting the sequential order of macitentan or placebo in Phase 1 and Phase 2, respectively: Group 1 = macitentan in Phase 1 then placebo in Phase 2; Group 2 = placebo in Phase 1 then macitentan in Phase 2. The local hospital pharmacy at the AUMC stored and dispensed the study medication and perform the administration of the drug accountability according to their local procedures. After 28 day treatment phase residual tablets were returned to the researcher. The number of residual tablets were recorded and destroyed according to procedures by the local hospital pharmacy.

Study treatment and follow-up

Patients were randomized to double-blind treatment with either 10 mg of macitentan once daily for 28 days, and subsequently placebo for 28 days, or vice versa. Treatment phases were followed by a 14-days wash-out period. macitentan as well as placebo were provided as blinded capsules. Background medication was recorded at the beginning of the study and maintained throughout the study. Throughout the study patients were asked to keep an angina diary that noted the frequency, duration and severity of anginal complaints. The study consisted of six visits, of which each consisted of reporting side-effects, Seattle Angina Questionnaire (SAQ), WHO performance status and an ECG. The SAQ is a self-administered questionnaire that measures the disease-specific severity of angina and its effect on quality of life and functioning [17]. Blood samples were taken at the first visit and at the third and fourth after the treatment phases.

Definitions.

The diagnosis of epicardial and microvascular CAS during acetylcholine spasm provocation was made in line with diagnostic criteria proposed by the Coronary Vasomotor Disorders International Study Group (COVADIS) group. Accordingly, all of the following were required for the diagnosis of epicardial CAS; (i) a reproduction of previously reported chest pain, (ii) induction of ischemic ECG changes, and (iii) >90% epicardial vasoconstriction by visual estimation [18], [19]. Microvascular CAS was diagnosed when the first two diagnostic criteria were met, but in the absence of epicardial vasoconstriction of >90% [19].

Outcomes

The primary outcome was the treatment induced change in anginal burden, which was calculated as the anginal burden during the medication phases (add-on macitentan and add-on placebo) minus the anginal burden during the run-in phase of the study. Anginal burden was calculated in two ways [1]: the duration (in minutes) * severity (on a Visual Analogue Scale (VAS) pain scale 1–10); and [2] as the frequency of angina attacks * severity (on a VAS scale 1–10).

The pre-specified secondary efficacy outcomes include [1] the change in patients reported outcomes measures via an angina diary (average VAS score, frequency and duration of anginal attacks per day) during the treatment phases (macitentan and placebo), and [2] change in the incidence and severity of anginal complaints as obtained by the Seattle Angina Questionnaire (SAQ) obtained at the end of the treatment phases (macitentan and placebo), and hospitalization for angina.

Assessment of the effect of antianginal drugs may preferably be measured by means of exercise capacity using standardised exercise testing, in which it is assumed that improved exercise capacity may account for benefit in terms of reduction of symptoms. In the VERA study, however, clinical evidence of symptomatic improvement in terms of anginal pain is measured by the SAQ and anginal burden as described above. Particularly in patients with vasospastic angina symptoms, whom often experience symptoms at rest, we believe the abovementioned outcomes more adequately reflect the benefit in terms of reduction of symptoms.

The pre-specified secondary safety outcomes include [1] detrimental changes in physical, laboratory or ECG parameters during medication use (macitentan or placebo) up to 2 weeks after discontinuation of the study medication, and [2] the occurrence of adverse events (i.e. hospitalization for anginal symptoms and) during the study period. Adverse events were assessed by a blinded investigator.

### 2.2 Statistical analysis

The VERA is a proof-of-concept study, therefore no formal sample size calculation was performed. We hypothesised that inclusion of 30 patients would allow to observe a difference between the efficacy of macitentan and placebo. Descriptive data are summarized as numbers with percentages for categorical variables, as mean with standard deviation for continuous variables with normal distribution and as median with (interquartile) range for continuous data with skewed distribution. Change in primary and secondary outcome measures in reaction to add-on treatment with macitentan and add-on placebo were compared using a paired T-test and Wilcoxon signed rank test. A p-value of 0.05 was considered statistically significant. All statistical analyses were performed using SPSS version 27 (IBM Corp., Armonk, NY, USA). A raincloud plot of the primary endpoint was made with an open access script on MatLab. [20].

## 3 Results

**Fig. 1:**
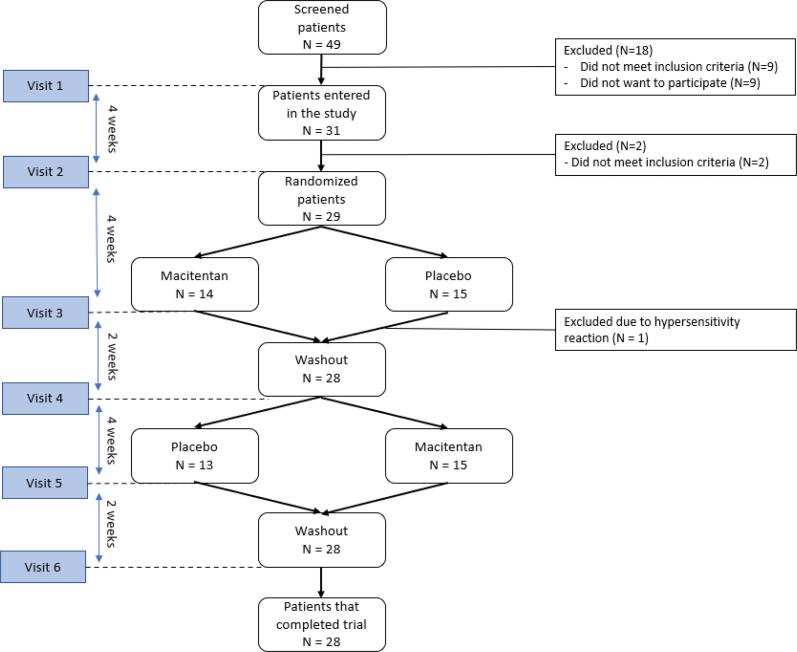
Flow chart of the study and included patients that were randomized.

Between November 2019 and July 2021 we enrolled 31 patients in to the VERA trial. Due to SARS-COVID19 pandemic inclusion of patients was temporarily halted between February 2020 and August 2020. The baseline characteristics as well as the background medication of the patients are described in Table 1. The mean age was 55.3 ± 7.6 years and the majority of patients were female (79%). Of the 31 patients who entered the study, 2 did not meet final inclusion criteria before randomization that did not allow further participation (see Fig. 1). Of the 29 patients that started on study medication, 28 patients completed both treatment phases of the study as one patient had a drug hypersensitivity reaction that prevented further participation. Background medication was maintained during the study with exception of one patient in who Atorvastatin was changed to Rosuvastatin in the run-in phase and in one patient the dosage of Diltiazem was increased from 200 mg to 300 mg between the third and the fourth visit (during wash-out period) by the treating physician.

**Table 1:** Patient baseline characteristics (n = 28).

**Demographics**	
Female	22 (79)
Age (years)	55.3 ± 7.6
BMI	25.7 ± 3.9
Heart rate (bpm)	64.5 ± 10.0
Blood pressure (mm Hg)	
Diastolic	76.0 ± 9.4
Systolic	118.9 ± 12.0
**Risk factors**	
Diabetes	1 (4)
Hypertension	7 (25)
Hypercholesterolemia	10 (36)
Family history of coronary artery disease	14 (50)
Smoking	
Ex-smoker	16 (57)
Current	1 (4)
Never	11 (39)
Any Alcohol consumption	15 (54)
Prior Myocardial infarction*	8 (29)
Prior PCI*	8 (29)
Prior CABG*	1 (4)
**Vasospasm endotype**	
Epicardial	19 (68)
Microvascular	9 (32)
**Symptoms**	
Resting angina	24 (86)
Effort angina	14 (50)
Dyspnoea	3 (11)
Concomitant medication during treatment phases	
Calcium channel blockers	20 (71)
Long acting Nitrates	16 (57)
ACE-inhibitors	5 (18)
Angiotensin receptor blockers	5 (18)
Statins	14 (50)
Aspirin	19 (68)
**Seattle Angina Questionnaire**	
Physical limitation	55.25 ± 19.92
Angina stability	45.54 ± 18.07
Angina frequency	33.21 ± 20.01
Treatment satisfaction	72.09 ± 15.73
Quality of life	38.84 ± 18.50
SAQ summary score	42.44 ± 13.31

PCI = percutaneous coronary intervention, CABG = coronary artery bypass graft, ACE-inhibitor = Angiotensin-converting enzyme inhibitor.

* all events were > 6 months ago.

Efficacy outcomes

**Fig. 2:**
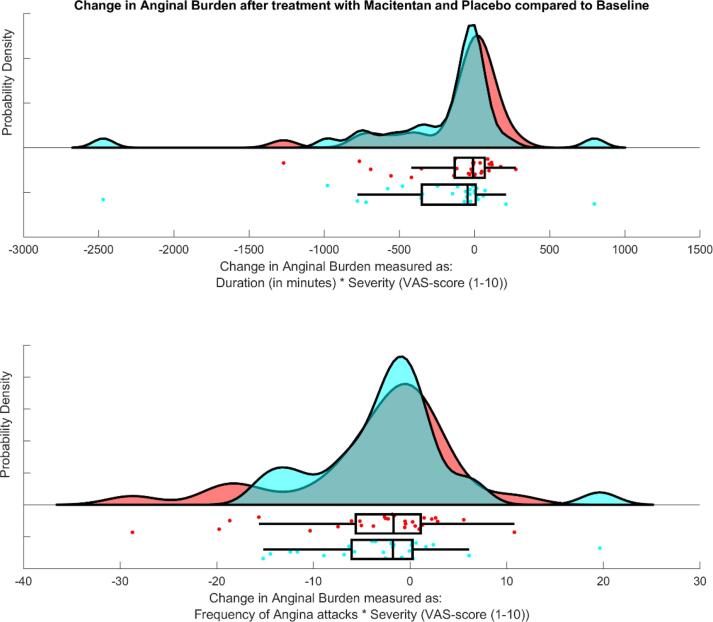
central figure: Raincloud plot of the effect of add-on treatment with placebo (blue) and Macitentan (red) on change in anginal burden comprising Gaussian kernel probability density, scatter and box-and-whisker plots. The top figure represents the change in anginal burden measured as the duration (in minutes) times the severity (on a 1–10 scale) and in the lower figure as the frequency of anginal attacks times the severity (on a 1–10 scale). (For interpretation of the references to colour in this figure legend, the reader is referred to the web version of this article.)

The primary efficacy outcomes are presented Table 2 and Fig. 2. The median change in anginal burden calculated as the duration * severity was −9 [Q1,Q3: −134 78] for macitentan versus −45 [Q1,Q3: −353 11] for placebo (p = 0.136) and the median change in the frequency of angina attacks * severity was −1.7 [Q1,Q3: −5.8 1.2] for macitentan versus −1.8 [Q1,Q3: −6.2 0.3] for placebo (p = 0.767).

**Table 2:** Primary and secondary endpoints.

	**Add-on Macitentan**	**Add-on Placebo**	**p-value**
*Primary outcomes*			
Δ Duration of angina per day (in minutes) * Severity (VAS score 1–10)	−9 [-134 78]	−45 [-353 11]	0.136
Δ Frequency of attacks per day * Severity (VAS score 1–10)	−1.7 [-5.8 1.2]	−1.8 [-6.2 0.3]	0.767
*Secondary endpoints*			
Δ Severity (VAS score 1–10)	−0.14 ± 1.44	−0.02 ± 1.57	0.246
Δ Duration of angina per day (in minutes)	−4.4 [-25.1 6.2]	−10.7 [-53.6 1.9]	0.199
Δ Frequency of attacks per day	−0.37 [-1.09 0.13]	−0.34 [-0.74 0.09]	0.432
Hospitalisation for angina	2	4	0.625
New onset ECG abnormalities	0	0	n/a

Δ represents the change from the run-in phase, VAS = visual analog scale, ECG = electrocardiography.

The secondary efficacy outcomes are presented in Table 2. The change in VAS score from the run-in phase did not differ between add-on treatment with macitentan and add-on treatment with placebo. The change in the average time of anginal complaints per day did not differ between add-on treatment with macitentan compared to placebo, nor did the reduction in number of attacks of day. No significant difference was observed in hospitalization for angina.

**Fig. 3:**
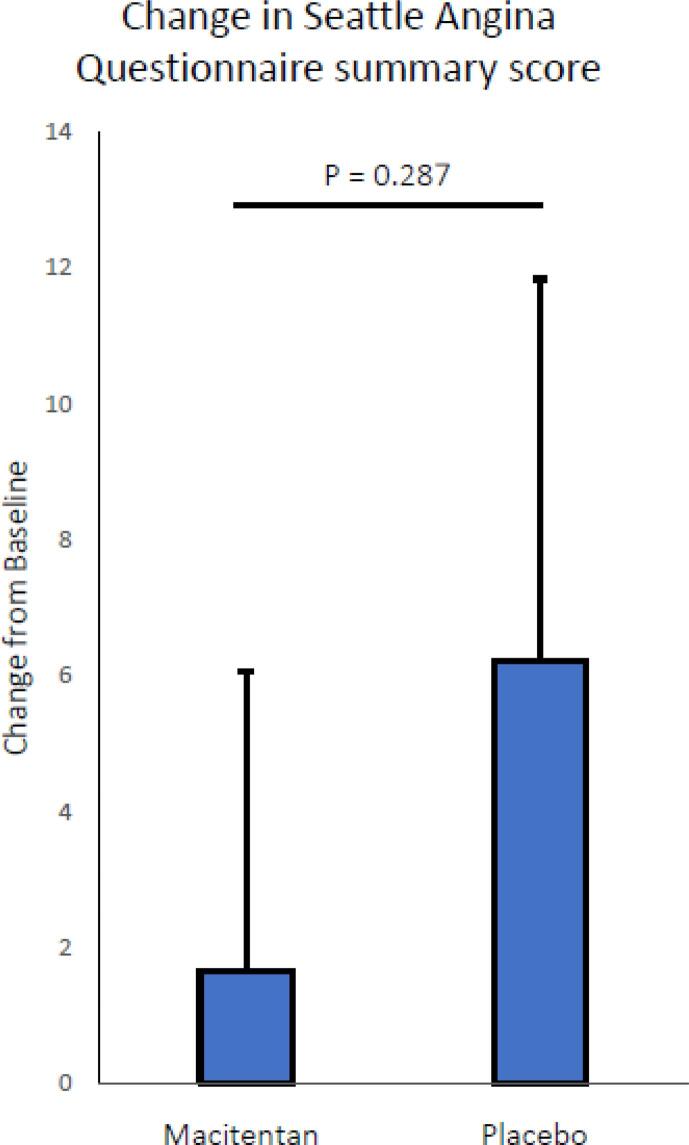
Mean (SE) change from baseline in Seattle Angina Questionnaire summary score after 4-week treatment with Macitentan and placebo. An increase in score represents an improvement in severity of angina and its effect on quality of life and functioning.

The outcomes of the SAQ summary score and the individual components of the SAQ can be found in Table 3 and Fig. 3. No significant difference between add-on treatment with macitentan compared to add-on treatment with placebo was observed in the summary score or in the scores of individual components of the SAQ. There was however a trend towards a reduced treatment satisfaction during the add-on treatment phase with macitentan (p = 0.076).

**Table 3:** Seattle Angina Questionnaire score.

	**Add-on Macitentan**	**Add-on Placebo**	**p-value**
Δ Physical limitation	−0.01 ± 15.19	5.23 ± 13.87	0.226
Δ Angina stability	9.82 ± 32.87	12.50 ± 33.68	0.792
Δ Angina frequency	0.36 ± 12.21	8.21 ± 21.61	0.112
Δ Treatment satisfaction	−3.79 ± 14.47	1.34 ± 12.19	0.076
Δ Quality of life	4.61 ± 18.40	5.21 ± 17.07	0.906
Δ SAQ summary score	1.65 ± 11.37	6.22 ± 14.49	0.287

An increase in score represents an improvement in severity of angina and its effect on quality of life and functioning. Δ represents the change from the run-in phase, SAQ = Seattle Angina Questionnaire.

At baseline, 10 (36%) patients were classified as WHO functional class II, 16 (57%) as III and 2 (7%) as class IV. After four weeks of add-on placebo 1 (4%) patient improved functional class, 23 (82%) remained the same and 4 (14%) deteriorated to a higher class and after four weeks of add-on macitentan 9 (32%) patients improved functional class, 12 (43%) remained the same and 7 (25%) deteriorated to a higher class. The change from baseline between add-on placebo and add-on macitentan did not differ significantly (p = 0.615).

Safety outcomes

The adverse events are summarized in Table 4. There were no apparent differences in incidence of self-reported adverse events between the treatment phases with macitentan and placebo except for the occurrence of peripheral oedema (7 during macitentan vs 1 during Placebo). One patient experienced a type II hypersensitivity reaction to macitentan (angioedema, pruritis and rash) whereafter participation in the study was discontinued. Finally, one patients developed hyponatraemia, after further investigation it was considered unrelated to treatment with macitentan and the patient continued the study medication. Laboratory parameters

**Table 4:** Adverse events during the treatment period and wash-out phase (total of 6 weeks).

	**Add-on Macitentan**	**Add-on Placebo**
Nasal congestion	2	2
Asthenia	3	5
Backpain	1	0
Confusion/ lack of mental clarity	1	0
Coronavirus-19 infection	1	0
Dry cough	1	0
Dizziness	1	3
Headache	2	4
Hypotension	1	1
Nausea	1	0
Stomach ache	0	1
Fluid retention	7	1
Palpitation	0	3
Hypersensitivity reaction (e.g., angioedema, pruritus, rash)	1	0

Changes in laboratory parameters from baseline in reaction to the treatment phases are shown in Table 5. Of note, change in NTpro-BNP was significantly decreased during treatment with macitentan compared to placebo (-25.30 ± 49.71 vs −1.04 ± 59.68, P = 0.032). Similarly, after treatment phase with macitentan a clinically non-significant decrease in hemoglobin, hematocrit, thrombocytes, leucocytes, alkaline phosphatase and NTproBNP, and an increase in ASAT was observed in the macitentan group that was not observed in the placebo group (all p < 0.05).

**Table 5:** Change in laboratory measurements.

	**Macitentan**	**Placebo**	P-value
Haemoglobin, mmol/L (n = 27)	−0.33 ± 0.40	0.05 ± 0.40	**<0.001**
Haematocrit, L/L (n = 27)	−0.01 ± 0.02	0.00 ± 0.02	**0.002**
Thrombocyte, 10^9/L (n = 27)	−11.67 ± 30.48	2.81 ± 20.19	**0.013**
Leukocyte, 10^9/L (n = 27)	−3.18 ± 11.13	−1.91 ± 11.11	**<0.001**
Creatinine, µmol/L (n = 27)	1.70 ± 5.59	0.74 ± 5.91	0.384
GFR, mL/min/1.73 m2 (n = 27)	−3.19 ± 8.57	−1.33 ± 7.63	0.140
Urea, mmol/L (n = 27)	0.02 ± 0.79	0.10 ± 0.84	0.546
NTpro-BNP, ng/L (n = 25)	−25.30 ± 49.71	−1.04 ± 59.68	**0.032**
Bilirubin, µmol/L (n = 26)	−0.73 ± 2.43	−1.04 ± 3.61	0.465
Alkaline phosphatase, µmol/L (n = 25)	−4.88 ± 11.88	1.00 ± 8.99	**<0.001**
Gamma-glutamyl transferase, U/L (n = 22)	1.36 ± 6.97	0.45 ± 3.65	0.508
AST, U/L (n = 26)	2.85 ± 5.70	0.65 ± 4.34	0.098
ALT, U/L (n = 27)	5.74 ± 12.51	−0.63 ± 6.46	**0.010**
high-sensitive cardiac troponin, ng/L (n = 17)	0.00 ± 2.26	−0.18 ± 2.35	0.668

GFR = glomular filtration rate, NTproBNP = N-terminal pro B-type natriuretic peptide, AST = aspartate aminotransferase, ALT = alanine aminotransferase.

## 4 Discussion

The VERA study is the first randomized, placebo-controlled clinical study to evaluate the clinical efficacy and safety of add-on macitentan in symptomatic patients due to epicardial or microvascular CAS despite background pharmacological treatment. The main findings of this study are that compared to placebo, macitentan [1]; id not effectuate a significant improvement in changes in anginal burden calculated as the duration * severity nor when calculated as the frequency of angina attacks * severity [2], did not significantly improve the average time of angina, number of attacks nor the average VAS score per day, and [3] did not result in a significant differences in the summary score of the SAQ nor its individual components. In the context of the current study, routine add-on treatment with macitentan in symptomatic patients due to epicardial or microvascular CAS does not effectuate improvement anginal symptoms.

In the VERA study we included symptomatic patient diagnosed with epicardial and microvascular CAS based on spasm provocation testing. Currently the term of vasospastic angina (VSA) is reserved for patients with epicardial vasospasm, whereas the term microvascular angina (MVA) includes vasoconstrictive disorders (e.g. microvascular vasospasm), and vasodilatory disorders that can be the result of endothelial dependent and endothelial independent mechanisms [18]. In the VERA study only patients diagnosed with VSA or MVA due to microvascular spasm were included as both disorders are the result of exaggerated vasoconstriction. In a previous study by Ford et al, a reduced vasorelaxation in response to acetylcholine and an increased vasoconstrictive response to ET-1 was found in VSA and MVA patients compared with control subjects [7]. This indicates a generalized systemic microvascular dysfunction in these patients wherein ET-1 acts as a potential mediator of endothelial dysfunction and exaggerated vasoconstriction. In a separate study the authors demonstrated that genetic dysregulation of endothelin-1 is implicated in MVA [21]. Although, in these studies MVA consisted largely of patients with an abnormal vasodilation in response to adenosine rather than an exaggerated vasoconstriction. Moreover, the vast majority of patients with VSA have shown to have co-existing MVA due to microvascular vasospasm [22], [23]. Subsequently, patients with solely vasodilatory disorders were not included as we did not expect a substantial therapeutic effect from short-term treatment (28 days) with macitentan.

In contrast to the VERA study, a small randomized, placebo-controlled study by Reriani, et al. evaluated the selective ETA-receptor antagonist, Atrastentan (10 mg o.d.) in 47 patients with MVA due to coronary microvascular endothelial dysfunction [11]. Microvascular endothelial dysfunction was defined as ≤ 50% increase in coronary blood flow (CBF) in response to the maximal dose of ACh compared with baseline CBF (impaired endothelial dependent vasodilation). After 6 months treatment, coronary microvascular endothelial function improved significantly compared to baseline in the Atrasentan group as versus the placebo group (ΔCBF: 39.67 % (23.23, 68.21) vs. − 2.22 % (−27.37, 15.28), P < 0.001). Unfortunately, following neutral results from phase III trials relating to the primary indications of Atrasentan (hormone-refractory prostate cancer) its production was discontinued, thereby stopping any further investigation of its potential application in MVA. Similarly, a small study in patients with coronary artery disease (n = 10) showed that a 60-minute infusion of ET receptor blockade improved coronary vascular function as the coronary flow reserve improved significantly in reaction to substance P, an endothelial dependent vasodilator. Furthermore, ETA blockade increased blood flow significantly more than did dual ETA/ETB blockade (p < 0.05) [24].

Currently, the PRIZE trial is enrolling [25], in which the efficacy and safety of adjunctive treatment with the selective ETA-receptor antagonist Zibotentan (10mgdaily) during a 12-weeks treatment period in patients with MVA that include vasoconstrictive disorders (microvascular vasospasm) and/or vasodilatory disorders (increased microvascular resistance or impaired coronary flow reserved). The results of this study can be expected 2023 and will hopefully clarify whether the potential beneficial effect of ETA-receptor antagonism is restricted to endothelial independent vasodilatory disorders.

**Fig. 4:**
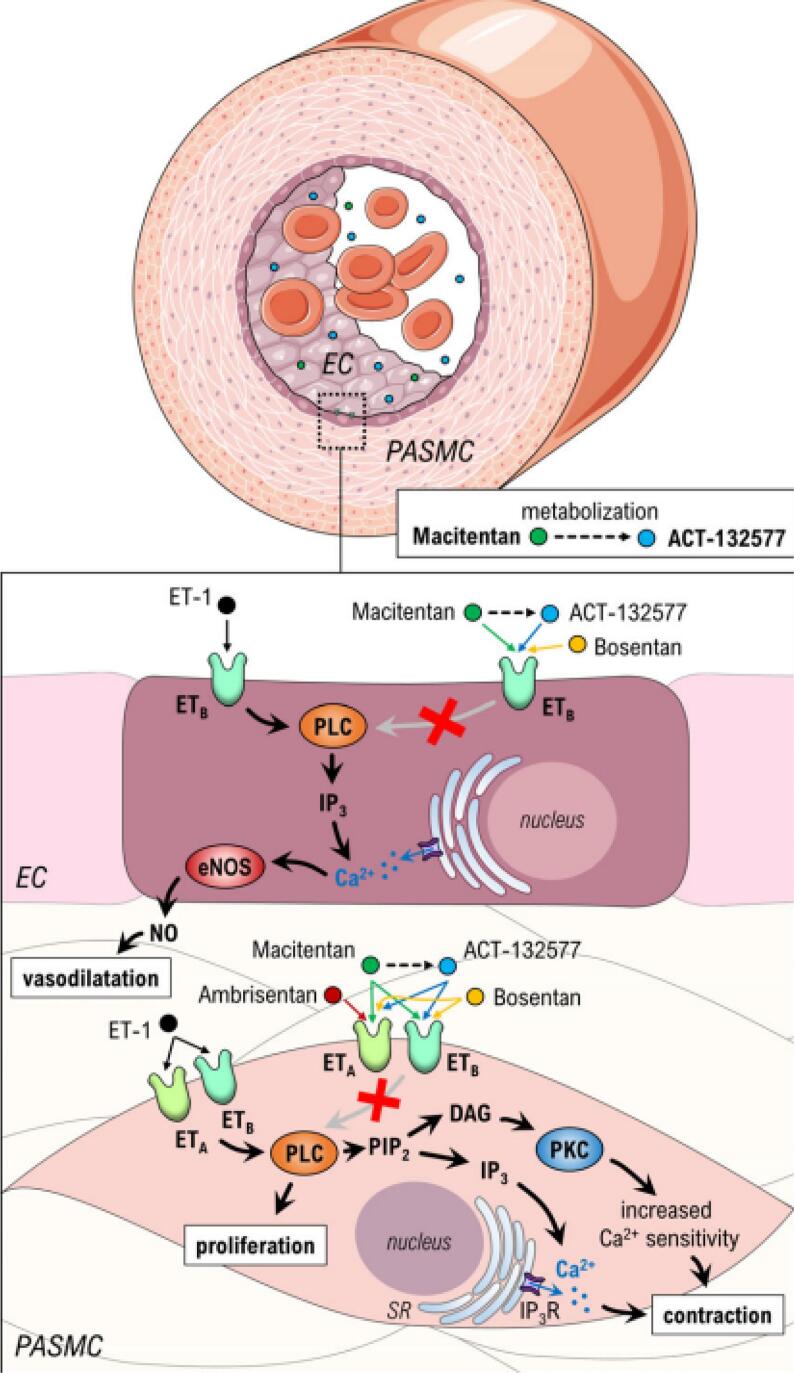
(copied from Basic & Clinical Pharmacology & Toxicology, 2018, 123, 103–113, Permission from authors/journal pending. Alternatively and if possible we would ask the journals graphic designers to reproduce a similar image.

The effect of ETA-receptor antagonism in CAS can be theorized to be twofold; directly though antagonism of exaggerated vasoconstrictive response of VSMC and indirectly through improvement of endothelial function. macitentan shows high affinity and long-term occupancy of the ET-A receptors in smooth muscle cells of the arteries. Thereby, macitentan prevents endothelin-mediated activation of second messenger systems that leads to vasoconstriction (Fig. 4). As the occupancy of ETA- receptors occurs rapidly after oral administration, it can be expected that macitentan rapidly affects the vascular tone of the coronary arteries. Additionally, the effect of ETA-receptor antagonism in CAS can be effectuated indirectly by increasing vasodilator function though improved endothelial function that counteracts on an exaggerated response of VSMC (Fig. 4). Most patients with CAS have concomitant endothelial dysfunction owing to the shared pathophysiological mechanisms [3], [26]. Improvement of endothelial function with ETA-receptor antagonism occurs both in patients with and without coronary artery disease [11], [24]. A treatment effect of endothelial receptor antagonism in CAS is therefore to be expected after long-term treatment as seen in other endothelial function modifying agents after long-term treatment in patients with CAS, such as with statins and ace-inhibiters [27], [28], [29].

A missing treatment effect of macitentan in the current study could therefore be due to the fact that the duration of treatment was not long enough for significant improvement in endothelial function as per rationale of the design of this study. A missing treatment effect of macitentan directly on VSMC could be because all the ETA-receptors on endothelial cells are occupied before macitentan can reach the ETA-receptor on the VMSC or that macitentan only induces vasodilation without preventing an exaggerated vasoconstrictive response. Therefore the dose of macitentan could not have been high enough in the current study to effectuate direct antagonism of VMSC. Furthermore, the results of the VERA trial were surprising as a unexpected placebo effect measured with careful documentation of symptoms in the angina diary was observed. The results might partially explained by the methodological rigor and careful conduction of the study, similarly as seen in the ORBITA trial [30].

macitentan is widely available and its safety has been documented extensively in numerous clinical trials investigating its effect on pulmonary hypertension. Most reported adverse events associated with macitentan in this patient group were headache, nasopharyngitis, and anemia [31]. Furthermore, the percentage of patients who discontinued the study drug owing to adverse events was substantial (10.7%). Remarkably, the occurrence and nature of self-reported adverse events in the current study in patients with CAS were closely similar between the treatment phase with macitentan and placebo. Although, the incidence of peripheral oedema was relatively more frequent during add-on treatment with macitentan which is most likely due to a cumulative effect of macitentan to the effect of other vasoactive medication. Of note, the occurrence of angina attacks that required hospitalisation was similar among both treatment phases. In one patient the study was discontinued after a confirmed hypersensitivity reaction to macitentan, which is reported to occur in 0, 1–1% of patients treated with macitentan. In our study no clinically significant changes in laboratory measurements were observed most likely due to the short exposure of the study drug. As a results of adverse events, the treatment satisfaction as assessed with the SAQ score was lower during the treatment phase with macitentan as compared to placebo.

## 5 Limitations

The limitations of the current study are due to the study design and include a relatively small number of patients. A particular concern of conducting a cross-over study is the possibility of a ‘carry over’ of treatment effect from one period to the next, however, there was a 14-days wash-out period between treatment phases, the duration of the study was short making it less likely that there was a large variation in anginal symptoms of the patients during the study period, the adherence was high (only 1 drop out), and the order of treatments phase was randomized. Quantifying the effect of macitentan with an angina diary in the current study was considered more appropriate than rechallenging the coronary circulation invasively with another vasospasm provocation test after 4 weeks of treatment. Furthermore, the protocol involved a relative short duration of treatment that may not have revealed all unexpected adverse reaction to be expected during long-term use. Another possible limitation is the use of study medication as add-on treatment to background medication. Background medication was maintained in nearly all patients throughout the study. As such any adverse events or clinical effect can be attributed to macitentan directly or indirectly. Our results may not apply to higher doses of macitentan or other ERA agents nor to different types of vasomotor dysfunction other than CAS for which future clinical studies are needed to assess the efficacy of pharmacological treatment with an ERA in these patients.

## 6 Conclusion

In symptomatic patients due to epicardial or microvascular coronary artery vasospasm despite background pharmacological therapy, 28 days of add-on treatment with the ET-1 receptor antagonist, macitentan did not reduce anginal burden compared to add-on treatment with placebo.

**Funding**

The VERA trial is an investigator-initiated clinical trial. This study was financially sponsored by Janssen-Cilag B.V.. Actelion Pharmaceuticals Nederland B.V. has provided the investigational medicinal product.

**Declaration of Competing Interest** Outside of the submitted work the authors disclose the following: P.D. has received consultancy fees from Philips and Abbott and research grants from Philips and Abbott. J.P. has received consultancy fees form Philips.

## CRediT authorship contribution statement

**Rutger G.T. Feenstra:** Conceptualization, Methodology, Investigation, Project administration, Data curation, Writing – original draft. **Tijn P.J. Jansen:** . **S. Matthijs Boekholdt:** Conceptualization, Methodology, Funding acquisition, Writing – review & editing. **Janet E. Brouwer:** . **Margriet I. Klees:** . **Yolande Appelman:** Investigation, Writing – review & editing. **Marianne E. Wittekoek:** Investigation, Writing – review & editing. **Tim P. van de Hoef:** Investigation, Writing – review & editing. **Robbert J. de Winter:** Writing – review & editing. **Jan J. Piek:** Writing – review & editing. **Peter Damman:** Writing – review & editing. **Marcel A.M. Beijk:** Conceptualization, Methodology, Funding acquisition, Investigation, Project administration, Data curation, Writing – original draft, Supervision.

## Declaration of Competing Interest

The authors declare that they have no known competing financial interests or personal relationships that could have appeared to influence the work reported in this paper.
